# Network pharmacology-based prediction and “gut microbiota-inflammation-brain axis” validation of the active ingredients and potential mechanisms of *Plantagins Herba* for treating diabetes-related cognitive dysfunction

**DOI:** 10.3389/fphar.2025.1601689

**Published:** 2025-06-20

**Authors:** Zhixuan Huang, Jian Liu, Hui Li, Yangwen Ai, Dongyue Zhou

**Affiliations:** ^1^ Jiangxi Province Key Laboratory of Traditional Chinese Medicine Pharmacology, Institute of Traditional Chinese Medicine Health Industry, China Academy of Chinese Medical Sciences, Nanchang, China; ^2^ Jiangxi Health Industry Institute of Traditional Chinese Medicine, Nanchang, China; ^3^ Institute of Chinese Materia Medica, China Academy of Chinese Medical Sciences, Beijing, China

**Keywords:** *Plantagins Herba*, Hispidulin, network pharmacology, diabetes-related cognitive dysfunction, “gut microbiota-inflammation-brain axis”

## Abstract

**Background:**

Diabetes-related cognitive dysfunction (DRCD) is increasingly recognized as a common complication. However, there are currently no specific remedies for DRCD. *Plantagins Herba* contains many active ingredients that can regulate blood lipids and blood glucose. It is used to treat cognitive impairment, but its therapeutic effects and molecular mechanisms on DRCD have not been reported.

**Purpose:**

To study the bioactive components, potential targets and molecular mechanisms of *Plantagins Herba* in the treatment of DRCD.

**Methods:**

Network pharmacology was applied to predict the active component of *Plantagins Herba* and the therapeutic targets of diabetes-related cognitive impairment. The molecular docking of the core components with the key targets was verified. Cell and animal models were established, and the mechanism by which hispidulin treats DRCD was explored via flow cytometry, Western blotting, behavioral experiments, HE staining, immunofluorescence, 16S rRNA and other techniques.

**Results:**

Based on the network pharmacology analysis, hispidulin derived from *Plantaginis Herba* was identified as a promising candidate for further investigation. The computational predictions suggest that the MAPK and PI3K/AKT signaling pathways may play pivotal roles in DRCD pathogenesis. *In vitro*, Hispidulin reduced inflammation and apoptosis in BV2 cells. It also improved the viability of HT22 cells under inflammation conditions and increased the expression levels of β-catenin and Cyclin D1 proteins. *In vivo*, hispidulin significantly reduced glucose and lipid metabolism disorders and the abundance of harmful flora in diabetic mice with cognitive impairment. The immunofluorescence results suggested that hispidulin reduced the activation of microglia in the mouse brain and decreased inflammation. The expression of p38MAPK/PI3K/AKT signaling pathway and β-catenin, Cyclin D1 protein, which confirmed regulatory effect of Hispidulin in hippocampal tissue.

**Conclusion:**

Hispidulin ameliorated disease manifestations in a DRCD-induced murine model, attenuating neuroinflammation and histopathological damage in hippocampal tissues through gut microbiota modulation.

## Graphical Abstract

**Figure:**
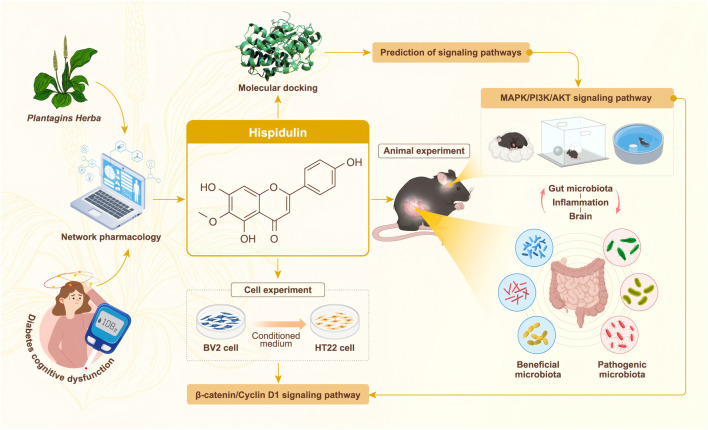
Network pharmacology-based prediction and “gut microbiota-inflammation-brain axis” validation of the active ingredients and potential mechanisms of Plantagins Herba for treating diabetes-related cognitive dysfunction.

## 1 Introduction

Diabetes is increasingly prevalent on a global scale, contributing significantly to the healthcare system’s burden. This rise in incidence not only impacts healthcare costs but also has serious ramifications for patients’ quality of life and life expectancy (Balooch Hasankhani et al., 2023). Among the numerous complications associated with diabetes, cognitive dysfunction stands out as a particularly concerning issue. Epidemiological studies indicate that individuals with diabetes have over a 50% greater likelihood of developing Alzheimer’s disease compared to their nondiabetic counterparts (Biessels and Whitmer, 2020). However, the specific mechanisms underlying cognitive impairment in diabetes remain unclear. Recent research suggests that insulin resistance and hyperglycemia may be involved in a number of possible reasons. Moreover, it is believed that vascular issues, oxidative stress, inflammation, and insulin resistance collectively play significant roles in the deterioration of cognitive function associated with diabetes (Verdile et al., 2015). Currently, the evaluation and management of cognitive impairment within diabetic patients mainly rely on methods that measure blood glucose levels, use magnetic resonance imaging, and use overall cognitive decline scales (Wu et al., 2023).

As diabetes advances, it significantly affects the integrity of the intestinal barrier, leading to changes in its permeability. The term “gut microbiota-inflammation-brain axis” usually means this situation when the intestinal barrier is compromised, as it allows for the translocation of intestinal bacteria and foreign endothelial proteins into the bloodstream and nervous system. The main nervous systems are affected by systemic inflammatory responses, which are triggered by this invasion (Wang et al., 2019). In the brain, neuroinflammation interferes with the ability of synapses, which impairs the function of neurons and eventually leads to the development of neurodegenerative disorders such Parkinson’s and Alzheimer’s diseases (Kölliker-Frers et al., 2021).

Plants of the *Plantago* family are a traditional Chinese medicine which have been used as folk medicine throughout the world (Samuelsen, 2000). *Plantagins Herba* is the dried whole grass of *Plantago asiatica L.* or *Plantago depressa Willd*, which belongs to the *Plantaginaceae* family. *Plantago asiatica L.* has been used as a vegetable and nutritious food in Asia for thousands of years. According to Traditional Chinese medicine it clears heat, disinhibits dampness, increases diuresis, frees stranguries, eliminates phlegm, cools blood, and detoxifies (Yang X. et al., 2020). Modern pharmacological studies have shown that the anti-diabetic effect of *Plantago asiatica L.* extract on type 2 diabetes rats may be related to the regulation of intestinal microbiota (Nie et al., 2019). Hispidulin is one of the flavonoid components of *Plantago asiatica L.* and has anti-inflammatory, anticancer, anti-oxidative, and anti-seizure qualities (Patel and Patel, 2017). In addition, Hispidulin is also effective as a 5-Lipoxygenase inhibitor (Adom et al., 2017). While hispidulin has shown potential benefits in diabetes treatment, research examining its influence on cognitive dysfunction related to diabetes remains lacking. Consequently, our study utilized network pharmacology predictions along with experimental verification to assess the effects of hispidulin on cognitive dysfunction associated with diabetes.

## 2 Materials and methods

### 2.1 Animals

Eight-month-old male C57BL/6 mice (20 ± 2 g body weight, n = 80) were purchased from Hunan SJA Laboratory Animal Co., Ltd. [license No. SCXK (Xiang) 2019–0004]. All animal experiments were checked and approved by the Laboratory Animal Ethics Committee of Jiangxi Health Industry Institute of Traditional Chinese Medicine (Jiangxi, Nanchang, China; animal ethics number: 2024007). The mice were kept in controlled environmental conditions, maintained within a temperature range of 22°–24°C and a relative humidity of 40%–60%. The mice were also given 12 h light/dark cycle which is very important for the healthy development of mice. Before the experimental procedures began, the animals were given a 1-week acclimatization period to adjust to their new environment. Throughout the acclimation phase, the animals were provided unrestricted access to food and water, ensuring their health and comfort in preparation for the upcoming experiments.

### 2.2 Cell lines and cell culture

BV2 and HT22 cells were purchased from Procell Life Science and Technology Co., Ltd. Both cell lines were maintained in Dulbecco’s modified Eagle’s medium (DMEM) supplemented with heat-inactivated 10% fetal bovine serum (FBS) and 1% penicillin/streptomycin solution (P/S) in a humidified atmosphere containing 5% CO_2_. These cells were subcultured at a dilution ratio of 1:4 every 3 days.

### 2.3 Reagents

DMEM, P/S, trypsin (HyClone, Utah, United States); FBS (Clark, VA, United States); 3-(4,5)-dimethylthiahiazo (-z-y1)-2,5-diphenytetrazoliumromide (MTT), lipopolysaccharide (LPS) (Solarbio, Beijing, China); Annexin V-Fluorescein isothiocyanate/Propidium Iodide (FITC/PI) apoptosis detection kit, nitric oxide (NO) assay kit, bicinchoninic acid (BCA) protein assay reagent kit (Beyotime, Shanghai, China); sodium dodecyl sulfate-polyacrylamide gel electrophoresis preparation kit (SDS-PAGE), electrochemiluminescence substrate kit (Epizyme, Shanghai, China); alloxan (Yuanye, Shanghai, China); metformin, donepezil, scopolamine hydrobromide polyvinylidene fluoride membrane (Merck, Darmstadt, Germany); insulin (INS) kit, low-density lipoprotein cholesterol (LDL-C) kit, triglyceride (TG) kit, total-cholesterol (T-CHO) kit (Jiancheng, Nanjing, China); animal tissue/cell total RNA extraction kit, all-in-one RT Supermix for qPCR, SYBR green qPCR master mix, p38MAPK (1:1,000), PI3K (1:1,000), AKT1 (1:1,000), β-catenin (1:500), Cyclin D1 (1:800) (Servicebio, Wuhan, China); β-actin (1:10,000), MultirAb HRP-Goat Anti-Rabbit Recombinant Secondary Antibody (H + L) (Proteintech, Rosemont, United States) were used in this study.

### 2.4 Network pharmacology analysis

#### 2.4.1 Database construction

Informations about *Plantagins Herba* were obtained from the Traditional Chinese Medicine Systems Pharmacology Database and Analysis Platform (TCMSP) database (https://tcmsp-e.com/). To identify the putative active components inside *Plantagins Herba*, a screening procedure was done based on specific criteria, which included an oral bioavailability ≥30% and a drug-likeness ≥0.18 (Ru et al., 2014). This initial filtration allowed for the selection of viable active ingredients that could be further analyzed (Ma et al., 2022). Once the active compounds were identified, they were submitted to the PharmMapper database (https://lilab.ecust.edu.cn/pharmmapper/) for an in-depth analysis of their corresponding biological targets. From this analysis, the top 30 potential targets associated with the active ingredients were selected for further evaluation. To ensure correctness and comprehensiveness, the protein names of these candidate targets were supplemented using information from the UniProt database (https://www.uniprot.org/). After that, the STRING database (https://cn.string-db.org/) and the UniProt database were used to transform the identified target proteins into the corresponding gene names. To specifically explore the connection between “cognitive dysfunction” and “diabetes mellitus,” the DisGeNET database (https://david.ncifcrf.gov/) was utilized with targeted searches for related terms. The goal of this procedure was to identify the targets that are connected to diabetes-related cognitive impairment. After identifying the relevant targets, any instances of duplication were meticulously removed to ensure the integrity of the data and the clarity of the results.

#### 2.4.2 Network establishment and analysis

(1) “Cognitive Dysfunction” and “Diabetes Mellitus”-related targets, (2) *Plantagins Herba* and (3) potential active components of *Plantagins Herba* and their corresponding targets were imported into Cytoscape 3.9.0 for data visualization and detailed analysis. The disease targets obtained from (1) and the component targets obtained from (2) were imported into the web tool Venny2.1.0. The corresponding database yielded the common targets, the intersection targets were imported into the STRING platform, and the restricted species was “*Homo sapiens*” with a confidence level of ≥0.9 to establish a protein‒protein interaction (PPI) network and export the TSV-formatted file. The TSV file was subsequently imported into Cytoscape 3.9.0 software, and the PPI network was topologically analyzed with degree >2 times the median, closeness centrality >1 times the median and betweenness centrality >1 times the median as the key targets. Moreover, the key targets associated with *Plantagins Herba*, notably those linked to “cognitive dysfunction” and “diabetes mellitus” were entered into the DAVID database. Here, Gene Ontology (GO) and Kyoto Encyclopedia of Genes and Genomes (KEGG) pathway enrichment analyses were performed. The analysis utilized “*Homo sapiens*” as the organism of interest and setting a significance threshold of “P < 0.05”. The findings from these analyses were visualized using the microbiology online mapping cloud platform (https://www.bioinformatics.com.cn/), which facilitated a clear presentation of the data. Finally, a compound-target-pathway network was constructed to correlate the active components of *Plantagins Herba* with the potential targets identified in the enrichment analysis of disease pathways. This comprehensive network was again imported into Cytoscape 3.9.0 software, which allowed for advanced visualization and detailed analysis of the intricate connections between the compounds, their targets, and the associated biological pathways (Subramanian et al., 2024).

### 2.5 Molecular docking

Through the use of molecular docking approaches, the study sought to investigate the possible interactions between hispidulin and three significant proteins, namely, AKT1, Caspase3 and MAPK1. To begin this process, the hispidulin molecule was sourced in a three-dimensional Structure Data File format from the PubChem database (https://pubchem.ncbi.nlm.nih.gov/). Subsequently, this molecule was converted into Protein Data Bank (https://www.rcsb.org/) format utilizing the PyMOL software, which is often used for visualizing and analyzing molecular structures. Following the conversion, the molecular structure was subjected to dehydration, a step which involves the removal of water molecules that may interfere with docking simulations. Additionally, any modified ligands that could affect the accuracy of the docking studies were also eliminated using the features available in PyMOL version 2.5.2. Once these preparatory steps were completed, the next phase involved the conversion of protein receptors associated with the active ingredients and target genes into pdbqt format. This conversion was accomplished using AutoDock Tools 1.5.6, which is specifically designed for preparing molecules for docking studies. Finally, the docking simulations were executed with AutoDock Vina version 1.1.2, enabling acomprehensive analysis of how the compounds bind to the receptor proteins associated with the target genes. Visualization and examination of these binding sites were then conducted with PyMOL 2.5.2, allowing for a thorough interpretation of the molecular interactions between hispidulin and the identified protein targets.

### 2.6 Effect of hispidulin on BV2 and HT22 cells

#### 2.6.1 Screening of the hispidulin concentration in BV2/HT22 cells

Exponentially proliferating BV2/HT22 cells, at a density of 1 × 10^4^ cells per well, were seeded in a 96-well plate and placed in an incubator until they reached a density of 80%. In this experimental setup, the control group received treatment with phosphate buffer solution (PBS), while other experimental groups were subjected to varying concentrations of the inducer, specifically at 12.5, 25, 50, and 100 µM. Following a culture period of 3 h, an aliquot of 50 µL of freshly prepared MTT solution at a concentration of 0.5 mg/mL was introduced into each well. After an additional incubation period of 3 h, the supernatant was carefully removed and 200 µL of dimethyl sulfoxide (DMSO) was added to each well to dissolve the formazan crystals. Subsequently, the samples were placed on a shaker for 3 h to assure total dissolution. Finally, the optical density of the resulting solution was measured at a wavelength of 540 nm microplate reader to assess the cellular viability.

#### 2.6.2 NO assay of BV2 cells

BV2 cells, at a density of 2 × 10^4^ cells per well, were plated in 96-well plates and subsequently cultured. For the experiment, the cells underwent pretreatment using varying concentrations of hispidulin, specifically 12.5, 25, and 50 μM for a duration of 1 h. After the pretreatment, the cells were subjected to LPS at a concentration of 100 ng/mL for 24 h. After the incubation period, the supernatants from the cell cultures were collected, and the levels of NO were assessed.

#### 2.6.3 Morphological observation of BV2 cells

The experimental grouping adhered strictly to the methodology outlined in Section 2.6.2. Upon the completion of the incubation period, the cells from each group were systematically examined and documented using microscopy techniques, capturing detailed images for further analysis.

#### 2.6.4 Analysis of BV2 apoptosis via flow cytometry

The experimental grouping adhered to the methodology outlined in Section 2.6.2. The precooled PBS was used to wash the cells three times after the incubation period was finished and the cell culture medium was carefully removed. The Annexin V-FITC was used to stain the cells in a dark environment after the washing procedure, resulting in the cell pellet being resuspended in binding buffer and then being stained with PI for accurate results. The samples were analyzed within 1 h after staining. Cells were gated based on forward scatter area and side scatter area to select the main population. Apoptotic cells at distinct phases were identified by Annexin V/PI staining using quadrant gates. The cells were measured by a Cyto FLEX flow cytometer (Beckman Coulter Company, United States).

#### 2.6.5 Reverse transcription real-time quantitative polymerase chain reaction (RT-qPCR) assay of BV2 cells

According to the kit instructions, each group of BV2 cells was extracted, reverse transcribed, and expanded. The 2^−△△Ct^ method was used to calculate the relative expression of the corresponding mRNA. The primer gene sequences were obtained from the NCBI database, and then provided to Shanghai Shenggong Bioengineering Co., Ltd. for design, synthesis and purification (Table 1).

**TABLE 1:** The primer sequences.

Gene (Mouse)	Primer sequences (5′ → 3′)
β-actin	Forward: CGT​GGG​CCG​CCC​TAG​GCA​CCA
	Reverse: TTG​GCC​TTA​GGG​TTC​AGG​GGG​G
Bax	Forward: GAA​CCA​TCA​TGG​GCT​GGA​CA
	Reverse: GCG​TCC​CAA​AGT​AGG​AGA​GG
Bcl2	Forward: ATG​CCT​TTG​TGG​AAC​TAT​ATG​GC
	Reverse: GGT​ATG​CAC​CCA​GAG​TGA​TGC
Caspase3	Forward: TGG​TTC​ATC​CAG​TCG​CTT​TG
	Reverse: ATT​CTG​TTG​CCA​CCT​TTC​GG
IL-1β	Forward: GAA​ATG​CCA​CCT​TTT​GAC​AGT​G
	Reverse: TGG​ATG​CTC​TCA​TCA​GGA​CAG
IL-6	Forward: CCC​CAA​TTT​CCA​ATG​CTC​TCC
	Reverse: CGC​ACT​AGG​TTT​GCC​GAG​TA
IL-10	Forward: GCT​CTT​ACT​GAC​TGG​CAT​GAG
	Reverse: CGC​AGC​TCT​AGG​AGC​ATG​TG
TNF-α	Forward: ACC​CTC​ACA​CTC​ACA​AAC​CA
	Reverse: ATA​GCA​AAT​CGG​CTG​ACG​GT

#### 2.6.6 Assays of the viability of HT22 cells

BV2 cells were grouped as follows: (1) Control group: with normal medium; (2) LPS group: with the addition of 100 ng/mL LPS; (3) 12.5 μM hispidulin group: with the addition of 12.5 μM hispidulin and 100 ng/mL LPS; (4) 25 μM hispidulin group: with the addition of 25 μM hispidulin and 100 ng/mL LPS; (5) 50 μM hispidulin group: with the addition of 50 μM hispidulin and 100 ng/mL LPS. After adding hispidulin for 1 h, the BV2 cells were treated with 100 ng/mL LPS for another 3 h. Next, the supernatant was collected to culture the HT22 cells. HT22 cells, at a density of 5 × 10^3^ cells per well, were plated in a 96-well plate, and the groups were the same as those described above. The culture medium from each group of BV2 cells was subsequently transferred to the HT22 cells, where they were incubated for 24 h.

#### 2.6.7 RT-qPCR and western blot analysis of HT22 cells

The experimental grouping used in this study adhered to the same methodology outlined in Section 2.6.6. The expression levels of IL-1β, IL-6, IL-10, and TNF-α mRNA in HT22 cells were detected by RT-qPCR, following the steps in Section 2.6.5. For Western blot, a protein sample of 30 μg was extracted from each experimental group and subjected to separation using sodium dodecyl sulfate-polyacrylamide gel electrophoresis. This process allowed for the effective resolution of the proteins, which were then transferred onto polyvinylidene difluoride membranes for further analysis. The membranes were treated with a blocking solution of skimmed milk in order to avoid nonspecific binding. Subsequently, the membranes were incubated with primary antibodies specific to the target proteins and were left at 4°C overnight to ensure adequate binding. After incubation, the membranes were washed to remove any unbound antibodies. Next, secondary antibodies, which are conjugated with detectable markers, were added to the membranes and incubated for a duration of 3 h. The membranes were imaged with chemiluminescence solution in the dark and visualized with a ChemiDocTM imaging system (Bio-Rad, United States).

### 2.7 Effect of hispidulin on cognitive dysfunction in diabetic C57BL/6 mice

#### 2.7.1 Animal modeling and drug administration

**FIGURE 1:**
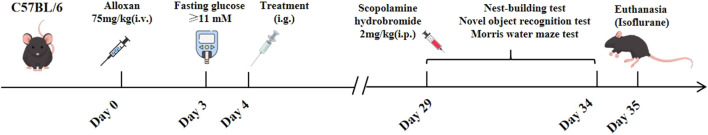
The process of animal experiments.

Based on their weight, mice were divided into two groups at random, with 10 mice in the control group and 10 in the diabetes-related cognitive impairment model group. Before beginning the experiment, the mice were fasted for 18 h. The next day, all of the mice were intravenously injected with alloxan (75 mg/kg) through the tail vein to induce a hyperglycemic model (Nordquist et al., 2008). The control group reveived a volume of normal saline injection through the tail vein. Blood was taken from the tail tips of the mice after 72 h of modeling, and the mice whose fasting glucose concentration was ≥11 mmol/L were considered successfully modeled. The successfully modeled mice were divided into 3 groups and gavaged for 26 days: the hispidulin group (5 mg/kg), the metformin + donepezil group (300 mg/kg + 1 mg/kg) (Hou and Li, 2021) and the model group (0.9% normal saline). The control group received 0.9% normal saline as well, and the body weights of the mice were recorded on a daily basis (Figure 1).

#### 2.7.2 Behavioral analysis

As part of this research, 0.9% normal saline was injected intraperitoneally into the control group of mice, which was used as a reference standard for comparison. In contrast, the other experimental groups received scopolamine hydrobromide (2 mg/kg) (Cheon et al., 2021) via intraperitoneal injection. This treatment was conducted 30 min prior to each testing session in order to effectively induce cognitive impairment associated with diabetes.

##### 2.7.2.1 Nest-building test

Each individual mouse was placed in a separate cage that contained soft paper to serve as nesting material. After allowing a 24-h period for acclimatization, the nesting behavior of the mice was carefully monitored and assessed. The evaluation of their nesting activities was based on established criteria found in academic literature. Specifically, a score of 1 point was assigned to mice that did not create any visible nest; a score of 2 points was given to those that formed an indistinct nest characterized by disorganized nesting materials that took up more than half of the cage space; a score of 3 points was obtained to mice that constructed visible nesting paper that occupies more than one-third but less than half of the cage; a score of 4 points was awarded to mice that constructed a complete nest, displaying a well-defined bowl shaped structure or nesting paper that occupies no more than one-third of the cage (Xiong et al., 2018).

##### 2.7.2.2 Novel object recognition test

The experimental setup for the novel object recognition task consisted of a rectangular enclosure and three distinct objects labeled as A, B, and C. Object C was intended to be significantly different from both A and B in order to make sure the mice could clearly distinguish it from the other two objects. In this configuration, objects A and B were identical, which provided a baseline for comparison. In the initial 2 days of the experiment, the mice were acclimated to the testing environment by spending 10 min in the enclosure to alleviate anxiety and become familiar with the surroundings. On the third day, the mice were placed in a box containing two identical objects A and B. They were allowed 5 min to freely explore these objects before being returned to their cages, providing a short break before the next phase of the experiment. After a 1-h interval, the setup was altered by substituting object B with the new object C, keeping the same spatial location for the replacement. The mice were then reintroduced into the box and permitted to explore once again for an additional 5 min. Ethanol was used to clean the area and objects between different testing phases to eliminate any potential olfactory cues that might affect the mice’s behavior. The data collected from the exploration sessions was analyzed using a recognition index, which was calculated by taking the ratio of the time of mice spent interacting with the novel object C to the total time spent engaging with both objects in the test. This method provided a measure of the mice’s ability to recognize and prefer the novel object, indicative of their memory and cognitive function (Yang et al., 2024).

Morris water maze test: The water maze consisted of a circular arena measuring 120 cm in diameter and 50 cm in height, featuring a platform with a diameter of 1 cm situated 1 cm below the water surface at the center of the first quadrant. This experiment is composed of three parts: (1) Positional experiment: This step took 5 days, with the mice practicing 4 times daily. The time to locate the underwater platform, swimming distance and trajectory were recorded with an infrared camera at the top. (2) Spatial exploration experiment: The underwater platform was removed on day 6, and the duration of swimming in the target quadrant was recorded over a 60-s period. (3) Visible platform experiment: After the spatial exploration test, the platform (1 cm above the water surface) was placed at the midpoint of the opposite quadrant, and the speed at which the mice reached the platform was recorded by a camera above the water maze (Serrano Sponton et al., 2018).

#### 2.7.3 Oral glucose tolerance test (OGTT) and serum biochemical test

The mice in each group were administered an oral gavage of 2 g/kg glucose, and blood glucose levels were assessed at 0,3,60,90 and 120 min using a glucometer (Yuyue, Jiangsu, China). The mice in each group were subsequently euthanized, and their serum was collected to determine the levels of fasting blood glucose (FBG), INS, LDL-C, TG, T-CHO, TNF-α and IL-6 via commercial kits.

#### 2.7.4 16S rRNA gene sequencing of the gut microbiota of mice

Sterile cryovials were used to collect fresh fecal samples from each group of mice. The fecal samples were then flash-frozen in liquid nitrogen for 1 h and subsequently transferred to −80°C for storage. The 16S rRNA gene sequencing of the intestinal microbiota was carried out by using the Biomarker Technologies Co., Ltd.

#### 2.7.5 Hematoxylin-eosin (HE) staining, immunofluorescence and immunohistochemistry in mouse brains

Whole-brain tissue from the mice was fixed with neutral buffered formaldehyde, and pathological changes in various regions of the brain were observed via HE staining. The microglia were labeled by Ionized calcium-binding adapter molecule 1 (IBA-1), and immunofluorescence was used to detect the activation of glial cells in the hippocampal CA1, CA3, and DG areas. Furthermore, immunohistochemistry was used to detect the expressions of p38MAPK proteins in mouse brain tissues.

#### 2.7.6 RT-qPCR detection of mRNA expression in the hippocampus of mice

RNA was extracted and detected from the hippocampal tissues of mice, and the mRNA levels of IL-1β, IL-6, IL-10 and TNF-α were analyzed.

#### 2.7.7 Protein expression levels in the hippocampus of mice

BCA was used to quantify the protein, which was done after the hippocampal region was lysed. The expression levels of p38MAPK, PI3K, AKT1, β-catenin, and Cyclin D1 in the hippocampus of mice from each group were assessed using Western blotting.

### 2.8 Data processing

All of the experiments were repeated at least three times. The data are expressed as the means ± standard deviations and were analyzed using GraphPad Prism 10.1.2. The mean values were analyzed by one-way analysis of variance or Student’s t test.

## 3 Results

### 3.1 Potential active ingredients and targets of *Plantagins Herba* for the treatment of diabetes-related cognitive dysfunction

**FIGURE 2:**
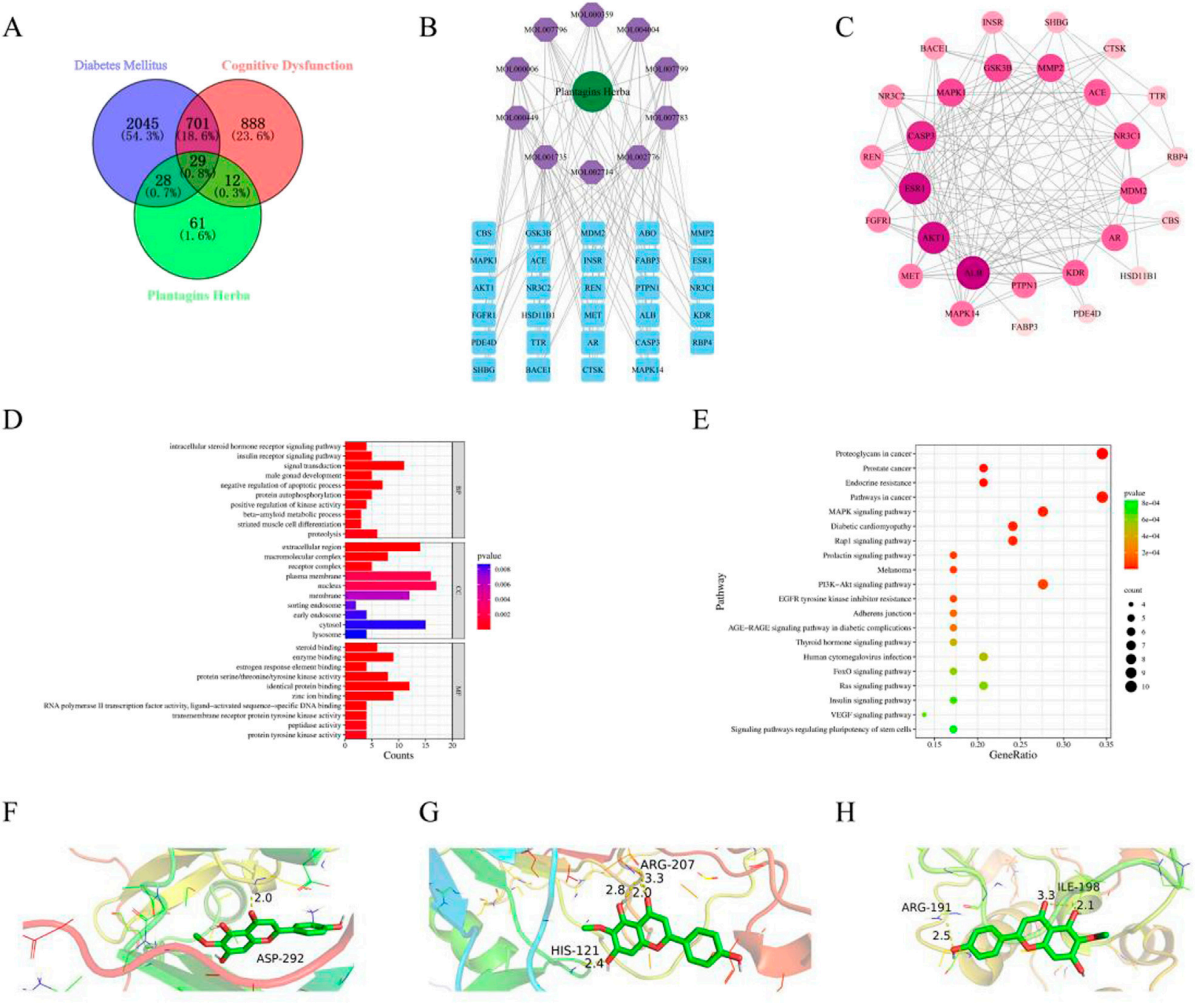
Network pharmacology and molecular docking. **(A)** Venn diagram of intersection targets of *Plantagins Herba*–diabetes mellitus–cognitive dysfunction. **(B)** Network of *Plantagins Herba*–active ingredients–potential target. **(C)** Intersection targets of *Plantagins Herba*–diabetes mellitus–cognitive dysfunction. **(D)** GO functional enrichment analysis of target genes for *Plantagins Herba* in the treatment of diabetes-related cognitive impairment. **(E)** KEGG analysis of core targets in the PPI network associated with the *Plantagins Herba*–DRCD. **(F)** Molecular docking model of AKT1 with hispidulin. **(G)** Molecular docking model of Capsase3 with hispidulin. **(H)** Molecular docking model of MAPK1 with hispidulin.

To elucidate the mechanism of *Plantagins Herba*, network pharmacology was used to screen all its chemical components through the TCMSP database by selecting standard parameters (oral bioavailability ≥30%, drug-likeness ≥0.18) (Table 2). The relevant structures of these components were entered into the PharmMapper database to determine which top 30 targets were present. The keywords “cognitive dysfunction” and “diabetes mellitus” were used to search the DisGeNET database, which ultimately yielded 4,433 disease targets related to DRCD. The intersection of drug targets, cognitive impairment targets, and diabetes targets yielded a total of 29 intersecting targets (Figure 2A). By matching the component targets with the disease targets, an herbal-compound-target network containing *Plantagins Herba*, 10 active compounds, and 29 candidate targets was established (Figure 2B). A visual network diagram was constructed from the saved TSV file via Cytoscape 3.9.0 software. A total of 41 hub targets for the therapeutic effect of *Plantagins Herba* on DRCD were identified. The protein interaction network suggested that these targets might be the hub targets through which *Plantagins Herba* affects drug efficacy in the treatment of DRCD (Figure 2C). For further study of the biological process and signaling pathway mechanism of *Plantagins Herba* in the treatment of DRCD, 41 hub targets of *Plantagins Herba* in the treatment of DRCD were uploaded to the DAVID database for GO and KEGG enrichment analyses. Terms were mostly associated with signal transduction, negative regulation of the apoptotic process, proteolysis, etc. According to GO analysis, it was revealed that the biological process (BP) terms were associated mainly with signal transduction, negative regulation of the apoptotic process, proteolysis, etc. Cellular component (CC) terms were mainly associated with the plasma membrane, nucleus, and extracellular region, etc. Molecular function (MF) terms were mainly associated with identical protein binding, zinc ion binding, and enzyme binding, etc. (Figure 2D). The top 10 enriched genes were subjected to KEGG pathway enrichment (Figure 2E). The top 20 signaling pathways associated with DRCD were visualized by ranking the p values. These pathways included the AKT signaling pathway and the MAPK signaling pathway. In molecular docking studies, a binding energy of less than −5.0 kcal/mol is generally regarded as a hallmark of strong binding affinity (Jaganathan and Kumaradhas, 2023). AKT1, Caspase3, and MAPK1 were molecularly docked with hispidulin, and the binding energies were all <−5.0 kcal/mol, indicating a strong binding capacity with all three proteins (Figure 2F-H).

**TABLE 2:** Potential active ingredients in *Plantagins Herba*.

Mol ID	Molecule name	OB (%)	DL
MOL001735	Dinatin/Hispidulin	30.97	0.27
MOL002714	baicalein	33.52	0.21
MOL002776	Baicalin	40.12	0.75
MOL000359	sitosterol	36.91	0.75
MOL004004	6-OH-Luteolin	46.93	0.28
MOL000449	Stigmasterol	43.83	0.76
MOL000006	luteolin	36.16	0.25
MOL007783	melampyroside	57.5	0.8
MOL007796	stigmasteryl palmitate	38.09	0.4
MOL007799	β-sitosteryl palmitate	30.91	0.4

### 3.2 Hispidulin protects BV2 and HT22 cells from damage caused by LPS

**FIGURE 3:**
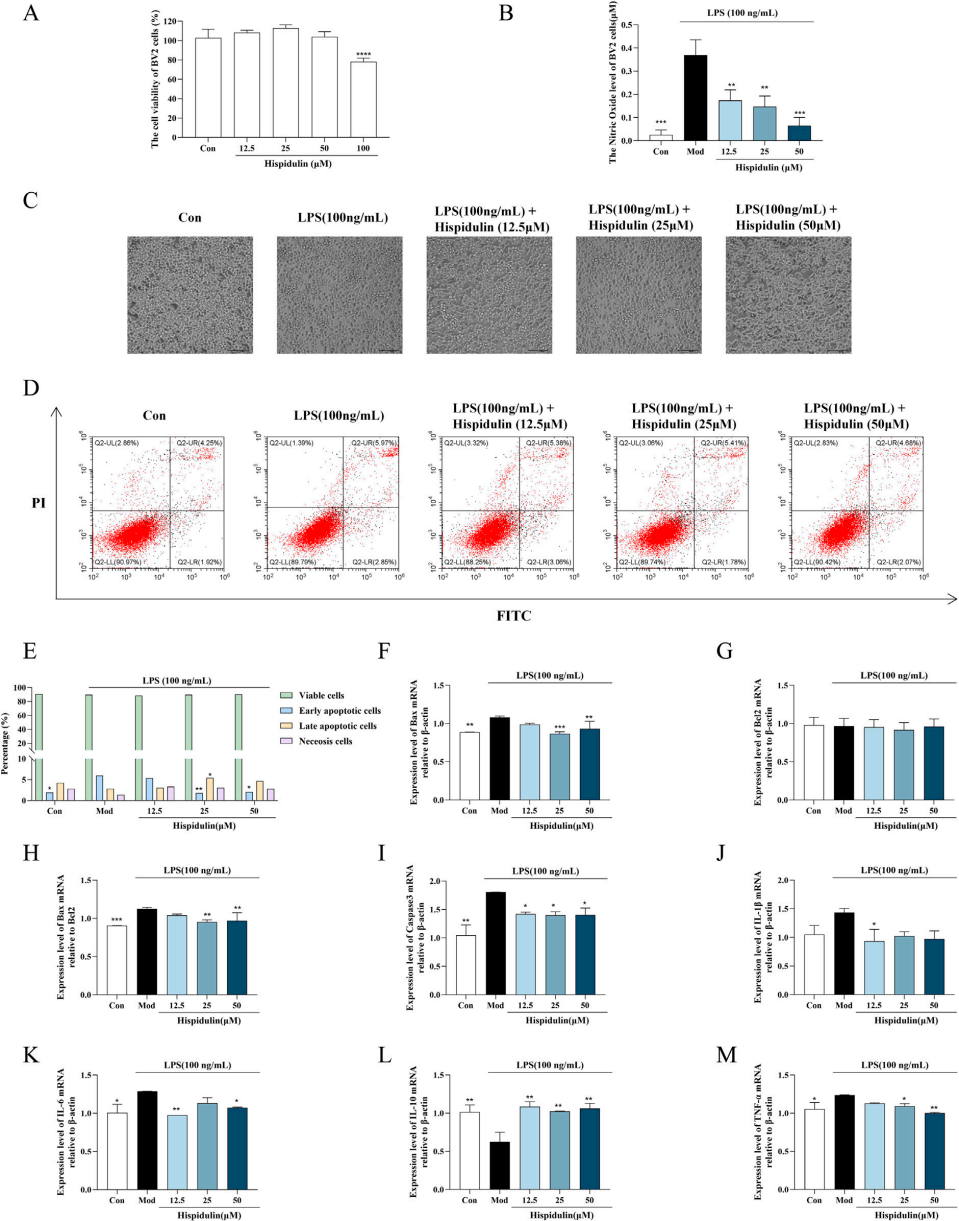
Effects of hispidulin on LPS-induced damage in BV2 cells. **(A)** Cytotoxicity of hispidulin on BV2 cells. **(B)** Effect of hispidulin on the relative level of NO in BV2 cells induced by LPS. **(C)** Morphology of the BV2 cells in each group. **(D)** Apoptosis of BV2 cells detected by flow cytometry. **(E)** Statistical analysis of the proportions of cells in different stages of apoptosis. **(F)** RT-qPCR analysis of Bax mRNA levels in BV2 cells. **(G)** RT-qPCR analysis of Bcl2 mRNA levels in BV2 cells. **(H)** RT-qPCR analysis of Bax/Bcl2 levels in BV2 cells. **(I)** RT-qPCR analysis of Caspase3 mRNA levels in BV2 cells. **(J)** RT-qPCR analysis of IL-Iβ mRNA levels in BV2 cells. **(K)** RT-qPCR analysis of IL-6 mRNA levels in BV2 cells. **(L)** RT-qPCR analysis of IL-10 mRNA levels in BV2 cells. **(M)** RT-qPCR analysis of TNF-α mRNA levels in BV2 cells. (The experiments were performed in triplicate, and the data are expressed as the means ± SDs, * *P*< 0.05, ** *P* < 0.01, *** *P*<0.001, **** *P*<0.0001).

**FIGURE 4:**
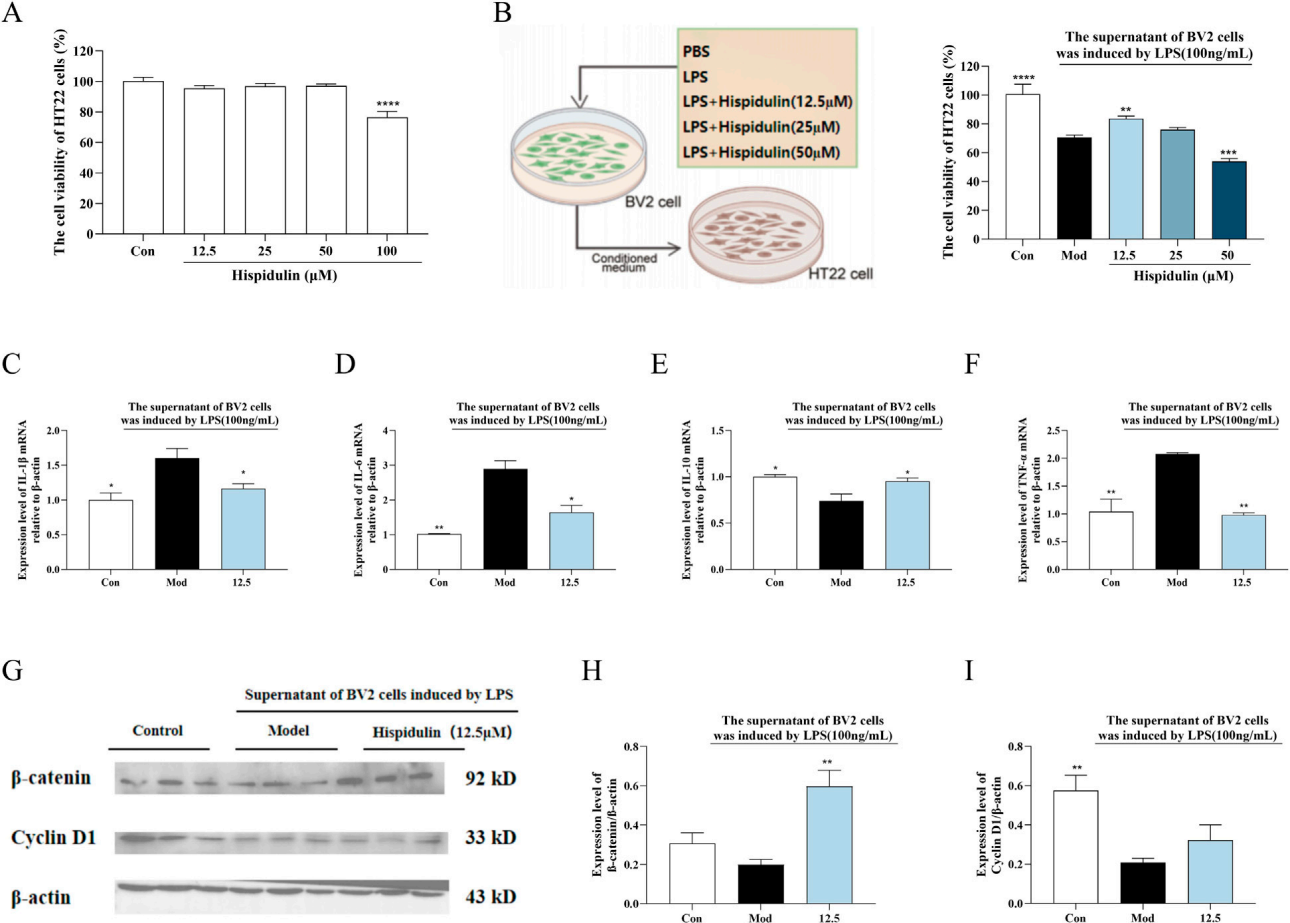
The effect of Hispidulin on LPS induced BV2 cell supernatant damage to HT22 cells. **(A)** Cytotoxicity of hispidulin on HT22 cells. **(B)** Cytotoxicity to HT22 cells of medium from BV2 cells treated with hispidulin. **(C)** RT-qPCR analysis of IL-Iβ mRNA levels in HT22 cells. **(D)** RT-qPCR analysis of IL-6 mRNA levels in HT22 cells. **(E)** RT-qPCR analysis of IL-10 mRNA levels in HT22 cells. **(F)** RT-qPCR analysis of TNF-α mRNA levels in HT22 cells. **(G)** The protein expression levels of β-catenin and Cyclin D1 in HT22 cells. **(H)** Statistical analysis of β-catenin levels in HT22 cells. **(I)** Statistical analysis of Cyclin D1 levels in HT22 cells. (The experiments were performed in triplicate, and the data are expressed as the means ± SDs, * *P*< 0.05, ** *P* < 0.01, *** *P*<0.001, **** *P*<0.0001).

To ensure the impact of various doses of hispidulin on the viability of both HT22 and BV2 cells, an MTT experiment was performed. When the highest concentration of hispidulin (50 μM) was applied to BV2 and HT22 cells, the viability of the cells was higher than 80%. This indicates that the concentration gradient of BV2 and HT22 cells was nontoxic, making them suitable for use in further activity studies (Figures 3A, 4A). To examine the effect of hispidulin on LPS, BV2 cells were treated with different doses of hispidulin for 1 h prior to stimulation with LPS for 24 h. In the control group, BV2 cells exhibited an epithelial cell-like morphology, with each cell having 2–3 protrusions that were relatively slender and a small cell nucleus. In contrast, the majority of the BV2 cells exhibited enlarged cell bodies, large round nuclei, prominent nucleoli, and short projections, all features typical of activated microglial cell morphology (Figure 3B). The synthesis of NO by LPS-stimulated BV2 cells was dramatically inhibited in a dose-dependent manner by hispidulin, as shown in the results of the NO test (Figure 3C). To further confirm the protective effect of hispidulin on LPS-stimulated BV2 cells, we performed Annexin V and PI double staining was performed by flow cytometric analysis. Hispidulin considerably decreased the LPS-induced apoptosis of BV2 cells in a dose-dependent manner (Figures 3D, E). The RT-qPCR experiments confirmed that hispidulin significantly reduced the mRNA levels of Bax, Caspase3, IL-1β, IL-6, TNF-α and increased the mRNA levels of IL-10 in LPS-induced BV2 cells (Figures 3F–M).

To determine the anti-inflammatory effects of hispidulin on the survival of hippocampal neurons in the presence of LPS-activated microglia, we conducted coculture experiments to simulate the coexistence of microglia and hippocampal neurons *in vivo*. The viability of the HT22 cells decreased when cultured in the medium from BV2 cells treated with LPS. When the HT22 cells were cultured in medium from BV2 cells treated with hispidulin (12.5 µM) and LPS, their viability increased (Figure 4B). Further analysis of β-catenin revealed that the expression level of Cyclin D1 protein expression level in HT22 cells decreased when they were cultured in the cell culture medium from BV2 cells treated with LPS. Hispidulin significantly reduced the mRNA levels of IL-1β, IL-6, TNF-α in HT22 cells, and increased mRNA level of IL-10 (Figures 4C–F). When HT22 cells were cultured with medium from BV2 cells treated with hispidulin and LPS, the β-catenin and Cyclin D1 protein expression levels in the HT22 cells increased, although they did not return to normal levels. These results indicate that hispidulin alleviates the damage caused to HT22 cells by neuroinflammation of BV2 cells (Figures 4G–I).

### 3.3 Hispidulin protects mice from damage caused by DRCD

#### 3.3.1 Effects of hispidulin on body weight and biochemical parameters in mice

**FIGURE 5:**
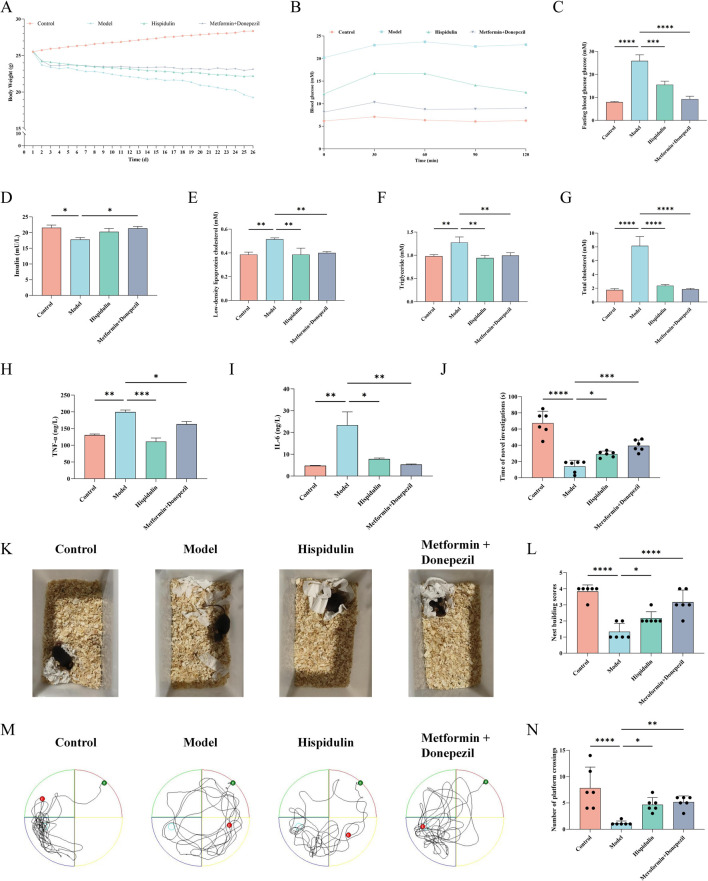
The effect of Hispidulin on biochemical indicators and behavior of mice in each group. **(A)** Body weights. **(B)** The curves of the OGTT tests.** (C)** FBG levels. **(D)** INS levels. **(E)** LDL-C levels. **(F)** TG levels. **(G)** T-CHO levels. **(H)** TNF-α levels. **(I)** IL-6 levels. **(J)** Statistics of the novel object recognition experiment. **(K)** Nest-building experiment. **(L)** Statistics of the nest-building experiment scores. **(M)** Trajectories of the mice in the water maze. **(N)** Statistical analysis of the results of the Morris water maze experiment. (The data are expressed as the means ± SDs, *P< 0.05, ** *P* < 0.01, *** *P*<0.001, **** *P*<0.0001).

Seventy-two hours after the intravenous injection of alloxan, all the mice exhibited symptoms of polyphagia, polydipsia, and polyuria. The mice used for modeling lost weight, and some of them even died. To confirm the effect of hispidulin on diabetes-related cognitive impairment in mice, relevant *in vivo* experiments were conducted. The results revealed that the body weights of the mice did not change significantly after hispidulin intervention, but their symptoms of thirst were alleviated (Figure 5A). After oral glucose administration, the mice in each group exprienced a sharp increase in serum glucose levels, reaching a peak at about 30 min and then gradually decreasing, as indicated by the OGTT data. The blood glucose level in the model group was significantly higher than that in the control group at each time point (Figure 5B). Hispidulin also significantly decreased the levels of FBG, LDL-C, TG and T-CHO (Figures 5C–F) while increasing the level of INS (Figure 5G). Hispidulin dramatically decreased the level of inflammatory factors TNF-ɑ and IL-6 in comparison to the untreated model group (Figures 5H, I).

#### 3.3.2 Effects of hispidulin on behavioral indices in mice

Nest-building tests are crucial for studying animal cognition and behavior. The Morris water maze is a common method used to assess an animal’s learning and memory abilities related to spatial location and orientation. In order to examine animal memory and learning ability, the novel object recognition test is frequently employed. As shown in Figures 5K, L the model group mice were unable to establish complete nests and had lower nest-building scores. After being treated with hispidulin, the mice were able to establish complete nests. In addition, hispidulin significantly increased the number of times that the mice investigated new objects (Figure 5J) and crossed the platform in the Morris water maze (Figures 5M, N).

#### 3.3.3 The effect of hispidulin on the diversity of gut bacteria in mice

**FIGURE 6:**
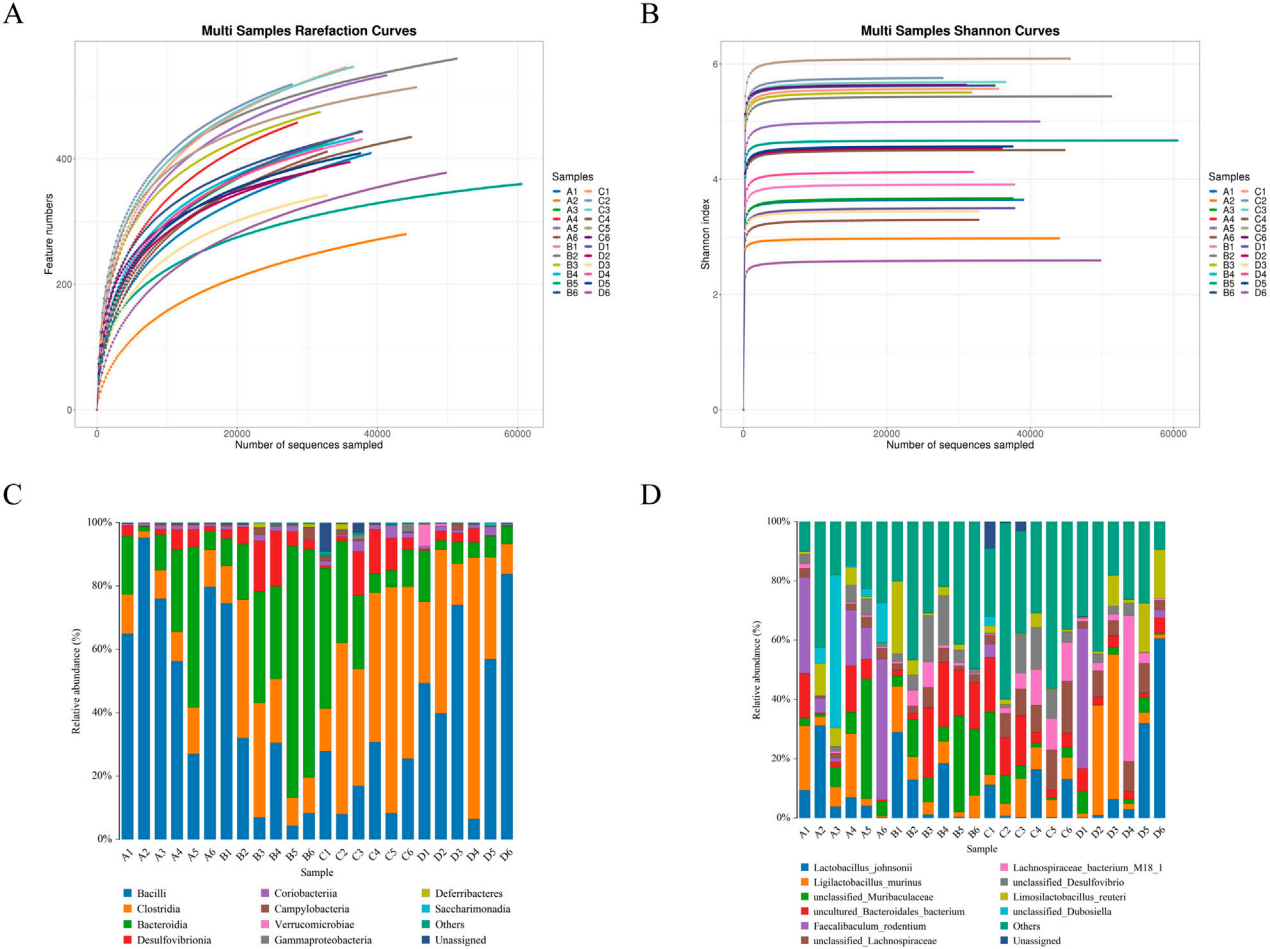
The effect of hispidulin on the diversity of gut bacteria in mice. **(A)** Dilution curve of the samples. **(B)** Shannon curves of the samples. **(C)** Structural graph of samples at the class level. **(D)** Structural graph of samples at the species level. (Control group: A1–A6; Model group: B1–B6; Hispidulin group: C1–C6; Metformin+Donepezil group: D1–D6).

A total of 1,251,243 CCS sequences were retrieved from 24 samples after sequencing and barcode identification, with each sample containing at least 40,940 CCS sequences and an average of 52,135 CCS sequences (Table 3). According to the results of the analysis, the sequencing depth of each sample was adequate and saturated, as shown by the Shannon and rarefaction analyses. Most of the microbial diversity information and the structure of the bacterial communities were reflected (Figure 6A, B). A comparison at the class level of the intestinal flora species structure of each group revealed that the levels of *Bacteroidia*, *Desulfovionia*, *Campylobacteria* and *Deferribacteres* in the hispidulin-treated group were lower than those in the untreated model group (Figure 6C). On the basis of the analysis of the structural differences in the intestinal flora among the groups at the genus level, a total of 10 genera were isolated from the fecal samples of each group at the genus level. Compared to the control group, the levels of *unclassified_Muribaculaceae* and *uncultured_Bacteroid_bacterium* were higher in the model group, but deareased after intervention with hispidulin (Figure 6D).

**TABLE 3:** Statistics of sample sequencing data processing results.

Sample ID	Raw CCS	Clean CCS	Effective CCS	AvgLen (bp)	Effective (%)
A1	52,970	52,952	43,029	1,468	81.23
A2	58,499	58,493	46,963	1,479	80.28
A3	50,375	50,337	41,134	1,459	81.66
A4	40,940	40,914	31,866	1,470	77.84
A5	52,462	52,437	43,885	1,460	83.65
A6	44,054	44,046	35,943	1,455	81.59
B1	50,518	50,480	40,868	1,484	80.9
B2	66,188	66,132	54,380	1,462	82.16
B3	47,221	47,193	35,204	1,462	74.55
B4	51,474	51,438	40,211	1,471	78.12
B5	64,314	64,073	61,327	1,459	95.36
B6	63,428	63,290	39,811	1,458	62.77
C1	53,033	52,996	39,839	1,460	75.12
C2	48,964	48,911	33,296	1,459	68
C3	51,778	51,744	40,294	1,463	77.82
C4	52,808	52,786	46,628	1,469	88.3
C5	59,085	59,058	48,343	1,461	81.82
C6	44,498	44,479	33,749	1,465	75.84
D1	50,979	50,954	41,710	1,456	81.82
D2	49,605	49,589	39,046	1,468	78.71
D3	42,304	42,286	35,266	1,480	83.36
D4	43,878	43,862	34,151	1,459	77.83
D5	48,412	48,386	40,327	1,476	83.3
D6	63,456	63,430	52,897	1,487	83.36

#### 3.3.4 The effect of hispidulin on the hippocampus in mice

**FIGURE 7:**
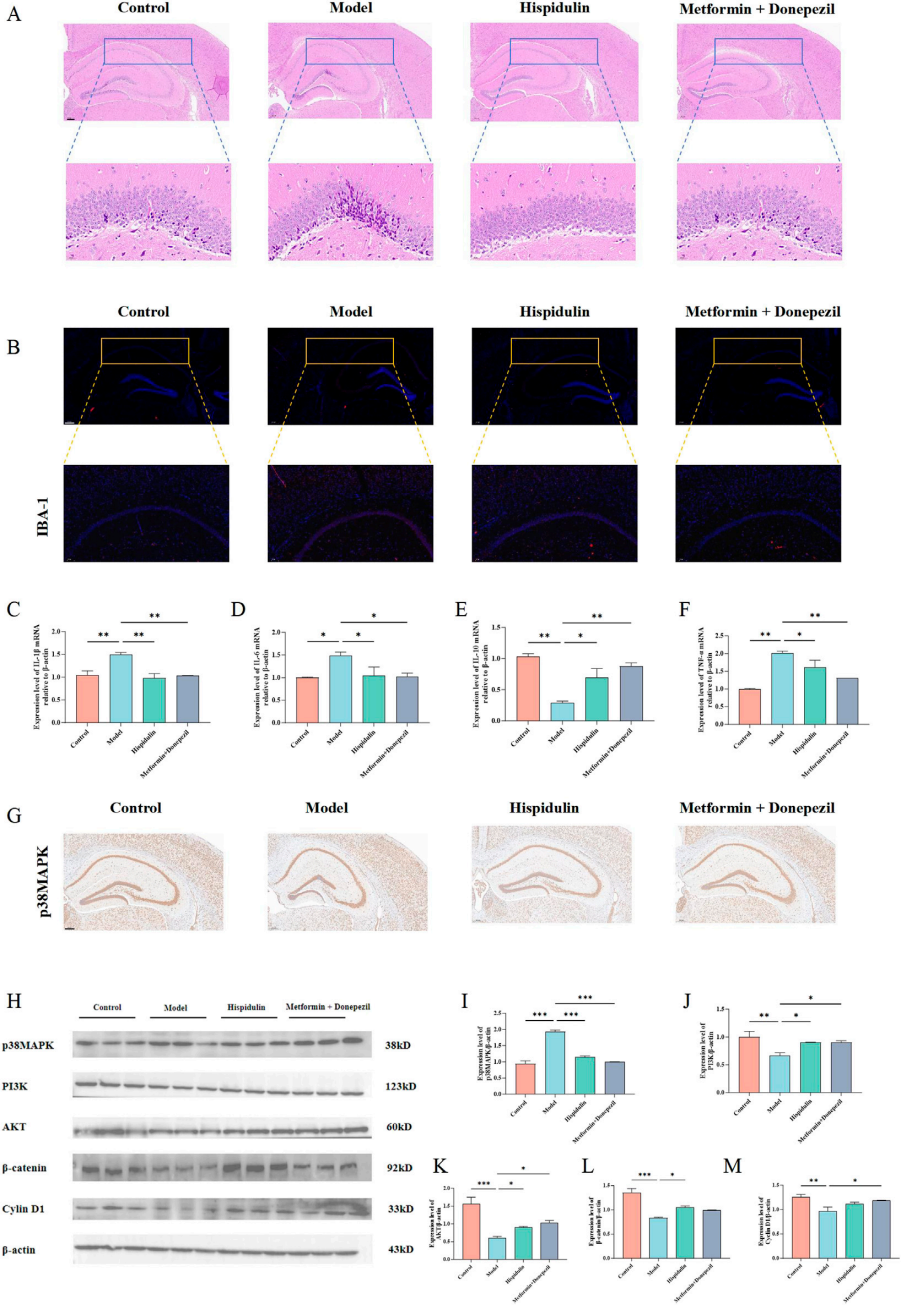
The effect of hispidulin on the hippocampus of mice in each group. **(A)** HE staining of mouse brains. **(B)** Immunofluorescence staining of mouse brains. **(C)** RT-qPCR analysis of IL-Iβ mRNA levels in the hippocampal tissue of mice. **(D)** RT-qPCR analysis of IL-6 mRNA levels in the hippocampal tissue of mice. **(E)** RT-qPCR analysis of IL-10 mRNA levels in the hippocampal tissue of mice. **(F)** RT-qPCR analysis of TNF-α mRNA levels in the hippocampal tissue of mice. **(G)** Immunohistochemical analysis of p38MAPK protein expression levels in the hippocampus of mice. **(H)** The protein expression levels of p38MAPK, PI3K, AKT, β-catenin and Cyclin D1 in the hippocampus of mice. **(I)** Statistical analysis of p38MAPK levels in the hippocampus of mice. **(J)** Statistical analysis of PI3K levels in the hippocampus of mice. **(K)** Statistical analysis of AKT levels in the hippocampus of mice. **(L)** Statistical analysis of β-catenin levels in the hippocampus of mice. **(M)** Statistical analysis of Cyclin D1 levels in the hippocampus of mice. (The experiments were performed in triplicate, and the data are expressed as the means ± SDs, * *P*< 0.05, ** *P* < 0.01, *** *P*<0.001, **** *P*<0.0001).

We performed HE and immunofluorescence staining on the brains of each group of mice were performed. Hispidulin restored the morphology of the hippocampal DG region (Figure 7A) and decreased the amount of activated microglia in the CA1 area (Figure 7B). Notably, marked pathological alterations were observed in cerebral tissues of model group mice, including disorganized neuronal architecture, vacuolar degeneration, and nuclear pyknosis. In contrast, Hispidulin treatment resulted in improved cellular alignment within hippocampal and cortical regions, accompanied by significant mitigation of neuronal damage. It also significantly reduced the mRNA levels of IL-1β, IL-6, TNF-α in the hippocampal tissue of mice and increased the mRNA level of IL-10 (Figures 7C–F). The results of immunohistochemistry suggested that hispidulin reduced the expression level of p38MAPK in the CA3 region of the hippocampal tissue of mice (Figure 7G). Furthermore, the protein expression levels of PI3K, AKT1, and β-catenin as well as Cyclin D1 were raised. Additionally, the protein expression levels of p38MAPK were decreased (Figures 7H–M).

## 4 Discussion

According to classic Chinese medicine, diabetes-related cognitive impairment is divided into three categories “Xiaoke Dian” (Liu X. J. et al., 2022). As the population ages, there has been a neglect of neurodegenerative diseases that manifest as progressive cognitive decline and memory impairment, both of which are complications linked to diabetes (Mayne et al., 2020). The concept of type 3 diabetes is gaining attention, highlighting the importance of proactive prevention and management of DRCD (Nguyen et al., 2020). Traditional Chinese medicine is employed for diabetes treatment due to its low toxicity and comprehensive regulatory effects (Tong et al., 2012). Research has indicated that some flavonoids have antidiabetic characteristics in animal studies. However, the exact targets of these flavonoids for enhancing blood sugar control remain unclear (Xu et al., 2014). This study utilzed network pharmacology in addition to experimental validation to explore the potential active components, targets, and mechanisms of action of *Plantagins Herba* in the treatment of DRCD. The analysis of the active ingredients network of *Plantagins Herba* as a potential target showed that hispidulin had the highest degree value. Through the PPI network of common targets, AKT1, Caspase3 and MAPK1 were identified as key targets. The GO analysis revealed the various effects of Plantaginis Herba. KEGG analysis showed that the MAPK and PI3K/AKT signaling pathways might have important roles in the effects of *Plantagins Herba* on DRCD. The binding energy between hispidulin and the core targets was then calculated by means of molecular docking. The results showed that the ligand and conformation are stable and suitable for further experimental verification.

Neuroinflammation stands out as a primary pathological mechanism underlying cognitive dysfunction associated with neurodegenerative diseases. This inflammatory response is significant in the progression of cognitive impairments, highlighting the urgent need to understand its contributions to neuronal health and disease (Adamu et al., 2024). Among the key players in this process is the transcription factor β-catenin, which is essential for promoting cellular proliferation. This function is largely mediated by β-catenin’s regulation of the target gene Cyclin D1 (Daisy Precilla et al., 2022). Recent studies have indicated that the expression of Cyclin D1 can be enhanced by the PI3K/AKT signaling pathway through the activation of β-catenin, illustrating a critical link between these biochemical pathways and cell growth. Additionally, experimental evidence has shown that the culture medium derived from LPS-stimulated BV2 cells negatively impacts the viability of SH-SY5Y cells, emphasizing the detrimental effect of neuroinflammation on neuronal cell health. This medium not only diminishes cell viability but also results in a notable reduction in the expression levels of both β-catenin and Cyclin D1 (Shakya and Chongthammakun, 2019). In light of these findings, we sought to integrate our prior network pharmacological predictions with empirical investigations using cell and animal models. This research aimed to evaluate the therapeutic potential of *Plantagins Herba* in treating cognitive dysfunction related to diabetes while also validating the predicted molecular targets associated with its efficacy. Through these combined methodologies, we can gain insights into the complex interactions that underpin cognitive decline in the context of diabetes and explore potential interventions. In order to replicate *in vivo* circumstances, BV2 and HT22 cells were cocultured in this work. The results showed that hispidulin reduced the inflammation, nitric oxide and apoptosis levels induced by LPS in BV2 cells. Furthermore, HT22 cells were exposed to the supernatant of BV2 cells that had been treated with hispidulin. Notably, HT22 cells exhibited and increased survival rate and enhanced expression of proteins in the β-catenin/Cyclin D1 pathway. According to these data, hispidulin controls the hippocampal neuron cell cycle to increase survival and lessens the inflammatory reaction of microglia.

The role that gut microbiota may play in the development of cognitive deficits related to diabetes has been emphasized by recent literature. This inflammatory condition appears to be initiated by alterations in the composition of gut microbiota (Zhang et al., 2021). In patients suffering from Alzheimer’s disease, studies have documented increased levels of *Bacteroides* within their microbiomes, which is indicative of a decrease in alpha diversity. Additionally, there has been a noted rise in the ratio of thick-walled *Bacteria/Bacteroides* phylum in experimental models of Alzheimer’s disease (Angoorani et al., 2022; Qiao et al., 2022). Furthermore, *Desulfovibrio* species have been recognized for their detrimental effects on the intestinal endothelium, as they contribute to a decrease in the integrity of this barrier and promote the maturation of microglia, primarily through the reduction of short-chain fatty acid levels (Liu et al., 2020). *Campylobacteriosis*, an infection resulting from *Campylobacter* bacteria, presents diarrhea as its principal clinical symptom (Yang H. et al., 2020). Research involving diabetic rats has shown a notable increase in the levels of *Campylobacter* in their intestinal microbiota, indicating a potential association between this infection and complications related to diabetes (Zhang et al., 2024). Moreover, the presence of *Deferribacteres* has been associated with type 2 diabetes mellitus, showcasing a clear positive relationship with FBG levels and pro-inflammatory cytokines (Nuli et al., 2019). When comparing type 2 diabetes patients to healthy individuals, it becomes evident that the abundance of *Deferribacteres* is markedly higher among diabetics, further establishing its positive correlation with both fasting blood glucose and inflammatory markers (Liu L. et al., 2022). The gut microbiota sequencing results of this study revealed that hispidulin reduced the levels of *Bacteroidia*, *Desulfovion*, *Campylobacteria* and *Deferribacteres* in mice at the class level and reduced the levels of *unclassified_Muribaculaceae* and *uncultured_Bacteroid_bacterium* in mice at the species level. These results suggest that hispidulin regulated the gut microbiota of mice, relieved the inflammatory response of the body and participates in the regulation of the course of DRCD.

Diabetes and obesity are conditions that exhibit low-grade inflammation, though the precise molecular mechanisms underlying this inflammation remain unclear (Cani et al., 2008). Insulin, a key hormone in the regulation of glucose levels, is known to activate both the MAPK pathway and the PI3K/AKT pathway. The MAPK/PI3K/AKT signaling pathway is crucial in insulin mediated regulation of glucose metabolism (Zhang et al., 2020). Furthermore, research has indicated that both the MAPK signaling pathway and the PI3K/AKT signaling pathway play significant roles in neuroinflammatory responses, highlighting their importance not only in metabolic processes but also in the nervous system (Feng et al., 2021). To further investigate the protective effect of hispidulin on DRCD, a mouse model of diabetes was untilized. The results showed that hispidulin significantly reduced the levels of FBG, LDL-C, TG and T-CHO in the mice. The ELISA data indicated that hispidulin similarly considerably lowered the amounts of TNF-α and IL-6 in the model mice. The results from the behavioral experiments showed that hispidulin significantly enhanced the nest-building score, the number of new objects explored, and the number of platform crossings in the Morris water maze. HE and immunofluorescence results suggested that hispidulin restored the morphology of the DG region in the mouse hippocampus and reduced the activation level of microglia in the CA1 region. The results of Western blotting demonstrated that hispidulin regulates signaling pathway proteins such as p38MAPK, PI3K, AKT1, β-catenin, and Cyclin D1. Activation of the MAPK pathway promotes the expression of cell cycle proteins such as cyclin D1 while concurrently suppressing the activity of apoptosis-associated proteins, thereby enhancing cellular proliferation and reducing apoptosis (Gu et al., 2022). Furthermore, the PI3K/AKT pathway reinforces proliferative signaling through crosstalk interactions with the MAPK pathway (Yang et al., 2010). Therefore, we hypothesize that the observed experimental results stem from the cross-regulatory interactions between the MAPK/PI3K/AKT and β-catenin/cyclin D1 signaling pathways. This integrated signaling mechanism promotes neuronal cell proliferation while attenuating apoptotic signaling pathways. In addition, evidence demonstrates that activation of the PI3K/Akt/mTOR signaling pathway enhances synaptic plasticity-associated protein synthesis and ameliorates cognitive dysfunction, as evidenced by recent mechanistic studies (Subramanian et al., 2022). These results indicated that hispidulin may reduce abnormal glucose and lipid metabolism as well as hippocampal inflammation in diseased mice. It suggested that hispidulin could act as a protective mechanism by activating the p38MAPK/PI3K/AKT signaling pathway to protect hippocampal neurons.

## 5 Limitations of the study

While the current study systematically elucidates the therapeutic mechanisms of *Plantaginis Herba* through the gut microbiota-inflammation-brain axis, several limitations should be acknowledged. Firstly, the temporal dynamics of gut microbiota remodeling during the intervention period warrant additional kinetic profiling to delineate cause-effect relationships. Secondly, our investigation primarily targeted the p38MAPK/PI3K/AKT signaling pathway. Thus, further exploration of potential interactions or crosstalk with other hippocampal plasticity-related pathways is necessary to establish a more comprehensive understanding of the underlying mechanisms and translational implications.

## 6 Conclusion

To summarize, our study demonstrated that Hispidulin reduced systemic inflammation and effectively alleviate cognitive dysfunction in diabetic mice by modulating the intestinal microbiota. This effect is associated with the activation of the p38MAPK/PI3K/AKT signaling pathway in the hippocampal tissue of the mice. These findings provide a robust scientific basis for the prospective clinical application of *Plantagins Herba* in treating cognitive deficits associated with diabetes.

Building upon the current findings, our subsequent investigations will focus on developing a colon-targeted prodrug of Hispidulin through computer-aided drug design. This strategy aims to enhance gut microbiota modulation while minimizing systemic exposure risks. The efficacy and microbiota-specificity of the prodrug will be rigorously evaluated using advanced 3D intestinal barrier models and organ-on-a-chip platforms. Concurrently, prodrug engineering will be employed to optimize pharmacokinetic profiles, thereby reducing first-pass metabolism and improving bioavailability. These endeavors will propel the paradigm shift in network pharmacology research from “multi-target prediction” to “spatiotemporal mechanistic verification,” offering methodological innovations for precisely deciphering the multi-component and multi-target mechanisms of traditional Chinese medicines.

## Data Availability

The original contributions presented in the study are publicly available. This data can be found here: Figshare, DOI: https://doi.org/10.6084/m9.figshare.29361896.

## Abbreviations

BCA, bicinchoninic acid; BP, biological process; CC, cellular component; DMEM, Dulbecco’s Modified Eagle’s Medium; DMSO, dimethyl sulfoxide; DRCD, diabetes-related cognitive dysfunction; FBG, fasting blood glucose; FBS, fetal bovine serum; FITC, Fluorescein isothiocyanate; GO, Gene Ontology; HE, Hematoxylin-Eosin; IB1-1, Ionized calcium-binding adapter molecule 1; INS, insulin; KEGG, Kyoto Encyclopedia of Genes and Genomes; LDL-C, low-density lipoprotein cholesterol; LPS, lipopolysaccharide; MF, Molecular function; MTT, 3-(4,5)-dimethylthiahiazo (-z-y1)-2,5-diphenytetrazoliumromide; NO, nitric oxide; OGTT, oral glucose tolerance test; P/S, penicillin/streptomycin solution; PBS, phosphate buffer solution; PI, Propidium Iodide; PPI, protein-protein interaction; RT-qPCR, real-time quantitative polymerase chain reaction; SDS-PAGE, sodium dodecyl sulfate-polyacrylamide gel electrophoresis; T-CHO, total-cholesterol; TCMSP, Traditional Chinese Medicine Systems Pharmacology Database and Analysis Platform; TG, triglyceride.
